# Telerehabilitation in Parkinson's disease: Influence of cognitive status

**DOI:** 10.1590/s1980-5764-2016dn1004012

**Published:** 2016

**Authors:** Alice Estevo Dias, João Carlos Papaterra Limongi, Wu Tu Hsing, Egberto Reis Barbosa

**Affiliations:** 1,2,3,4 Hospital das Clínicas da Faculdade de Medicina da Universidade de São Paulo, SP, Brazil.

**Keywords:** Parkinson's disease, cognition, voice treatment, telerehabilitation

## Abstract

**Background:**

The need for efficacy in voice rehabilitation in patients with Parkinson's disease is well established. Given difficulties traveling from home to treatment centers, the use of telerehabilitation may represent an invaluable tool for many patients.

**Objective:**

To analyze the influence of cognitive performance on acceptance of telerehabilitation.

**Methods:**

Fifty patients at stages 2-4 on the Hoehn-Yahr scale, aged 45-87 years old, with cognitive scores of19-30 on the Mini-Mental State Examination, and 4-17 years of education were enrolled. All patients were submitted to evaluation of voice intensity pre and post in-person treatment with the Lee Silverman Voice Treatment (LSVT) and were asked to fill out a questionnaire regarding their preferences between two options of treatment and evaluating basic technological competence.

**Results:**

Comparisons between pre and post-treatment values showed a mean increase of 14dBSPL in vocal intensity. When asked about potential acceptance to participate in future telerehabilitation, 38 subjects agreed to take part and 12 did not. For these two groups, 26% and 17% self-reported technological competence, respectively. Agreement to engage in remote therapy was positively associated with years of education and cognitive status.

**Conclusion:**

Responses to the questionnaire submitted after completion of traditional in-person LSVT showed that the majority of patients (76%) were willing to participate in future telerehabilitation. Age, gender, disease stage and self-reported basic technological skills appeared to have no influence on the decision, whereas other factors such as cognitive status and higher school education were positively associated with acceptance of the new therapy approach.

## Abstract (pt)

**Embasamento:**

A eficácia na reabilitação da voz em pacientes com doença de Parkinson está bem estabelecida. Tendo em vista as dificuldades de lidar com a locomoção de casa para centros de tratamento, o uso da telerreabilitação pode representar uma ferramenta inestimável para muitos pacientes.

**Objetivo:**

Analisar a influência do desempenho cognitivo na aceitação da telerreabilitação.

**Métodos:**

Participaram cinquenta pacientes em estágios 2-4 de acordo com a escala de Hoehn-Yahr, com idade entre 45 e 87 anos, escores cognitivos de 19 a 30 no Mini-Exame do Estado Mental e escolaridade entre 4-17 anos. Todos foram submetidos à avaliação da intensidade da voz antes e depois do tratamento pelo *Lee Silverman Voice Treatment* (LSVT) e foram convidados a responder um questionário sobre suas preferências entre duas opções de tratamento.

**Resultados:**

O tratamento resultou em aumento médio de 14dBNPS. Quando questionados sobre a possibilidade de aceitação para participar de um futuro programa de telerreabilitação, 38 indivíduos concordaram e 12 não. Em relação a estes dois grupos, a competência tecnológica foi referida em 26% e 17%, respectivamente. A aceitação à telerreabilitação foi positivamente relacionada com anos de estudo e estado cognitivo.

**Conclusão:**

As respostas ao Questionário após a conclusão do LSVT tradicional mostraram que a maioria dos pacientes (76%) concordaria em participar de uma futura telerreabilitação. Idade, sexo, estágio da doença ou competência tecnológica não pareceu influenciar na adesão à telerreabilitação enquanto que outros fatores, como estado cognitivo e anos de escolaridade foram positivamente relacionados com a aceitação da nova forma de terapia.

## INTRODUCTION

Speech and voice disorders in Parkinson's disease (PD) are classified as hypokinetic dysarthria and characterized by gradual deterioration of intelligibility of verbal communication.^1^ Common findings include abnormal sensory processing, neuropsychological abnormalities, reduced loudness, monopitch, monoloudness, reduced stress, breathy or hoarse voice quality, imprecise articulation, short rushes of speech and hesitant or nonfluent speech.^2-6^ Dysarthria affects nearly 90% of PD patients^7^ and is particularly incapacitating due to worsening of social interactions^8^ and interference with activities of daily living.^9-10^ There appears to be a correlation between the degree of dysarthria and other factors such as motor status, disease progression and cognitive functions.^11-12^ It is estimated that less than 5% of PD patients have engaged in speech rehabilitation,^13^ the most common reasons for non-adherence being physical limitations, lack of companion, long travel distances and financial costs.^14^

The introduction of new technologies has allowed the development of new approaches to treatment such as remote rehabilitation or *telerehabilitation.*^15-18^ Preliminary studies comparing efficacy of in-person versus remote treatment of speech therapy in PD disclosed similar results.^20-21^ Moreover, speech telerehabilitation in PD might offer additional advantages such as accessibility and opportunity for those living far from treatment centers and for those having difficulty in locomotion.^22^ On the other hand, there appears to be some factors that might limit adherence to new technologies and the identification of some of these factors may help to determine how to employ the best practices available. The aim of the present study was to evaluate the influence of cognitive function on adherence to telerehabilitation for speech treatment in PD.

## METHODS

**Participants.** Fifty patients diagnosed with PD were enrolled. Participants met the following inclusion criteria: diagnosed with PD according to the UK Parkinson's Disease Brain Bank Criteria,^23^ stage 2 to 4 according to the Hoehn & Yahr (H&Y)^24^ modified scale and the presence of voice and speech complaints. Exclusion criteria were: previous surgery for PD, dementia as assessed by the Mini-Mental State Examination^25^ (MMSE <24) and the Informant Questionnaire on Cognitive Decline in the Elderly (IQCODE <3),^26^ language disturbances and previous or concomitant speech therapy. All subjects were asked to sign an informed consent form to participate.

**Procedures.** All procedures were performed during the "on" phase and consisted of the following:

*Neurologic examination.* Subjects were submitted to a comprehensive neurologic examination including MMSE and H&Y scale before beginning treatment.

*Speech and voice evaluation.* For each subject, the initial evaluation was performed before the first treatment session and the final assessment after the last session. Individual evaluations took 30 minutes and consisted of computerized acoustic analysis of voice intensity (acoustic correlate of vocal loudness) by VoxMetria version 4.7 (CTS Informatics) installed on a Macbook pro Apple (16GB RAM, HD 500GB, i7). The voice signal was captured by a Lesson unidirectional microphone HD 74 , connected to the computer and placed at a distance of 30cm away from the mouth. In Voice Analysis mode, an isolated and sustained vowel /a/ emission was recorded. Subjects were asked to sit still and to perform the vowel emission for as long as possible. Results were extracted from the Statistical Function of the program in dBSPL (sound pressure level) units. Initial and final recordings were discarded in order to minimize irregularities.

*Speech rehabilitation.* All subjects were individually submitted to the Lee Silverman Voice Treatment (LSVT or LSVT LOUD). This was a one-month program comprising 16 sessions over a four-week period. Each session had a mean duration of one hour.^27^

*Questionnaire.* At the end of the rehabilitation program, subjects received detailed information about the speech rehabilitation process and filled out a structured questionnaire to evaluate their impressions about the in-person rehabilitation, telerehabilitation and technological competence (Table 2).

**Table 2:** Responses for in-person rehabilitation, remote rehabilitation acceptance and technological competence.

**Question 1: How did you feel participating in this in-person study?**			**Question 2: Would you agree to participate in remote therapy using computer and internet?**		
**Answers**	**N**	**%**	**Answers**	**N**	**%**
a. Satisfied	50	100	a. Yes	38	76
b. Not satisfied	0	0	b. No	12	24
**Question 3: For those answering “yes” to question #2: do you consider yourself skilled for computer and internet use?**			**Question 4: For those answering “no” to question #2: do you consider yourself skilled for computer and internet use?**		
**Answers**	**N**	**%**	**Answers**	**N**	**%**
a. Yes	10	26	a. Yes	2	17
b. No	28	74	b. No	10	83

*Statistics.* Descriptive statistics included percentage, mean and standard deviation. Correlations among clinical variables and the opinion of participants were determined based on Spearman´s analysis. A value of 0.05 (α=5%) was established for rejection of the null hypothesis.

**Ethics.** The present study was approved by the Ethics Commission for Analysis of Research Projects (CAPPesq) of the Administration of Hospital das Clínicas da Faculdade de Medicina da Universidade de São Paulo (HCFMUSP), nº 841/11.

## RESULTS

Sixty-nine subjects were initially selected to take part in the study. Nineteen (27.5%) of these were subsequently excluded for not being able to complete the entire rehabilitation program due to non-adherence. Reasons for dropping out included socio-economic factors, physical constraints (pain, malaise, freezing) and lack of companion to attend sessions. Fifty patients fully participated and their general characteristics are shown in Table 1. Comparisons between pre and post values show a mean increase of 14dBSPL in vocal intensity for the sustained vowel assessment. Table 2 shows the degree of satisfaction regarding face-to-face rehabilitation. When asked about potential acceptance to participate in a future telerehabilitation program, 38 subjects agreed to take part and 12 did not. For these two groups, 26% and 17% self-reported technological competence, respectively. Statistical correlations are shown in Table 3. Individual opinions did not correlate with gender, age or stage of the disease. Significant differences were found between opinions, years of education and cognitive status.

**Table 1:** Demographics and clinical presentation.

	Value
Female n (%)	26 (52)
Male n (%)	24 (48)
Age, mean ± SD (range)	73±8.45 (45-87)
H&Y, mean ± SD (range)	3±0.66 (2-4)
MMSE, mean ± SD (range)	27±1.22 (24-30)
Years of education, mean ± SD (range)	10±4.34 (4-17)
Vocal intensity, mean before/after treatment	61/75

H&Y: Hoehn&Yahr; MMSE: Mini-Mental State Examination.

**Table 3:** Correlations between remote therapy acceptance and clinical and demographic data.

Characteristics	Correlation coefficient	Spearman´s analysis p ≤ 0.05
Acceptance × gender	+0.061	p = 0.821
Acceptance × age	+0.273	p = 0.634
Acceptance × H&Y	+0.364	p = 0.070
Acceptance × MMSE	+0.405	p = 0.013
Acceptance × years´ education	+0.382	p = 0.018

H&Y: Hoehn&Yahr; MMSE: Mini-Mental State Examination.

## DISCUSSION

A combination of motor (rigidity, bradykinesia, tremor) and non-motor (neuropsychiatric, sensory, autonomic) features of PD may result in a characteristic speech and voice disturbance known as hypokinetic dysarthria.^28^ While the efficacy of pharmacological and surgical approaches is limited and controversial, the benefits of speech therapy are well established.^29-33^ LSVT is the gold standard for voice rehabilitation in PD and is structured based upon concepts involving motor learning, acquisition of new abilities and neuroplasticity. As originally conceived, LSVT is performed in a person-to-person approach and its effectiveness is widely recognized.^34^ Recently, researchers have taken advantage of new technologies and the combination of the LSVT concept with broadband internet connections, known generally as telerehabilitation, has been tested with favorable results. Despite the effectiveness of the method, many patients are reluctant to adhere to a treatment program for a number of reasons, including physical limitations, geographical factors and social or family constraints.^35^ In the present study, subjects with PD and voice symptoms were submitted to LSVT as a first-choice treatment.^36,37^ As expected, results demonstrated significant improvement in voice intensity and intelligibility in accordance with previous studies^38,39^ and reflected the general satisfaction of our patients with the clinical results. In this context, patients were further asked about their opinions about engaging in a future project involving remote rehabilitation at their homes, regardless of their skills in dealing with computers and the internet. The general willingness to participate in a telerehabilitation program appears to indicate that factors such as independence, comfort and cost reductions with transportation and travels may have a significant impact on treatment adherence. On the other hand, some patients chose not to participate in a remote rehabilitation program, where reasons given included not having transportation problems, the need to establish closer face-to-face contact, and the opportunity to spend time outside the home environment. A greater proportion of patients refusing the remote therapy considered themselves unskilled in basic technological knowledge and this could be another reason for non-adherence although this factor did not appear to significantly influence the decision process. Nevertheless, a previous assessment of basic computer knowledge should precede indication of telerehabilitation and efforts should always be made to recruit the help of family members or caregivers.

In the present study, gender, age and disease stage appeared to have no influence on adherence to remote therapy and this finding was in accordance with previous studies focusing on factors that could influence acceptance of telerehabilitation.^40-42^ Patients with advanced PD and the elderly might be less skilled and face some difficulties in dealing with new digital technology but may benefit considerably from remote therapy, which could help overcome difficulties with locomotion and transportation. Thus, this group should be encouraged to participate in such treatment programs.^43^ On the other hand, younger patients are expected to be familiar with digital technologies and more prone to engage in a new treatment program regardless of physical limitations.

Our results suggest that level of education and MMSE scores may influence adherence to telerehabilitation. In fact, higher-educated subjects with tend to acquire new knowledge in a more appropriate way and a correlation between level of education and MMSE scores has been reported.^44,45^ In the present study, establishing a cut-off level for the MMSE of >24 did not exclude the possibility that many of our patients may have presented with subtle cognitive impairment that are often encountered in PD patients even at early stages.^46,47^ We recognize that the MMSE is a poor predictor of cognitive status in PD, as it does not evaluate certain cognitive domains such as visuospatial orientation, non-verbal memory and executive functions known to be impaired in PD. In the present study, the correlations between adherence and specific domains of cognitive functions were not explored, where only total MMSE score was considered as a means of excluding overt dementia. Thus, it may well be the case that discrete limitations regarding perception, comprehension, retention or visuospatial orientation acting to reduce the ability to adapt to new technologies could have been missed. Further studies utilizing more sophisticated tools to evaluate specific domains in PD and their potential impact on treatment adherence are currently underway.

Ideally, therapeutic planning should consider unlimited access to specialized care for all PD patients seeking voice rehabilitation and recent studies have reported that remote therapy can be considered a useful alternative.^48,49^ Decisions regarding treatment options should take into account a number of variables including the level of effort (physical, emotional, cognitive) necessary to engage in face-to-face therapy or, alternatively, in remote therapy.^50^ Additional factors not addressed in the present study are of fundamental importance and should be investigated in further studies, including potential comorbidities (visual or hearing impairment, abnormal postures, poor manual dexterity), technological infrastructure offered by the therapist (enabling privacy and confidentiality) and basic prerequisites expected of patients (emotional and psychological aspects, interest, basic knowledge).

## References

[1] Darley FL, Aronson AE, Brown JR (1975). Motor speech disorders.

[2] Berardelli A, Rothwell JC, Thompson PD, Hallet M (2001). Pathophysiology of bradykinesia in Parkinson's
disease. Brain.

[3] Ho AK, Iansek R, Bradshaw JL (1999). Regulations of parkinsonian speech volume: the effect of
interlocutor distance. J Neurol Neurosurg Psychiatr.

[4] Ho AK, Bradshaw JL, Iansek T (2000). Volume perception in parkinsonian speech. Mov Disord.

[5] Sapir S, Ramig L, Hoyt P, O'Brien C, Hoehn M (2002). Phonatory-respiratory effort (LSVT^®^) vs
respiratory effort treatment for hypokinetic dysarthria: comparing speech
loudness and quality before and 12 months after treatment. Folia Phoniatr.

[6] Dias AE, Limongi JCP (2003). Treatment of vocal symptoms in Parkinson's disease: the Lee
Silverman method. Arq Neuropsiquiatr.

[7] Ramig LO, Fox C, Sapir S (2008). Speech treatment for Parkinson's disease. Expert Rev Neurother.

[8] Fox CM, Ramig LO (1997). Vocal sound pressure level and self-perceptions of speech and
voice in men and women with idiopathic Parkinson disease. Am J Speech Lang Pathol.

[9] Ma EPM, Yiu EMI (2001). Voice activity and participation profile: assessing the impact of
voice disorder and daily activities. J Speech Lang Hear Res.

[10] Kuopio AM, Marttila RJ, Helenius H, Toivonen M, Rinne UK (2000). The quality of life in Parkinson's disease. Mov Disord.

[11] Dias AE, Barbosa MT, Limongi JCP, Barbosa ER (2016). Speech disorders did not correlate with age at onset of
Parkinson's disease. Arq Neuropsiquiatr.

[12] Miller N, Noble E, Jones D, Allcock L, Burn DJ (2008). How do I sound to me? Perceived changes in communication in
Parkinson's disease. Clin Rehabil.

[13] Ramig LO, Fox C, Sapir S (2008). Speech treatment for Parkinson's disease. Expert Rev Neurother.

[14] Dias AE, Limongi JCP, Barbosa ER, Hsing WT (2016). Voice telerehabilitation in Parkinson's disease. Codas.

[15] Hart J (2010). Expanding access to telespeech in clinical settings: inroads and
challenges. Telemed J E Health.

[16] Hailey D, Roine R, Ohinmaa A, Dennett L (2011). Evidence of benefit from telerehabilitation in routine care: a
systematic review. J Telemed Telecare.

[17] Mashima PA, Doarn CR (2008). Overview of telehealth activities in speech-language
pathology. Telemed J E Health.

[18] Brown J (2011). ASHA and the evolution of telepractice. Perspect Telepract.

[19] Cherney LR, van Vuuren S (2012). Telerehabilitation, virtual therapists and acquired neurologic
speech and language disorders. Semin Speech Lang.

[20] Reynolds AL, Vick JL, Haak NJ (2009). Telehealth applications in speech-language pathology: a modified
narrative review. J Telemed Telecare.

[21] Grogan-Johnson S, Alvares R, Rowan L (2010). A pilot study comparing the effectiveness of speech language
therapy provided by telemedicine with conventional on-site
therapy. J Telemed Telecare.

[22] Dias AE, Limongi JCP, Hsing WT, Barbosa ER (2013). Digital inclusion for telerehabilitation speech in Parkinson's
disease. J Parkinsons Dis.

[23] Hughes AJ, Daniel SE, Ben-Shlomo Y, Lees AJ (2002). The accuracy of diagnosis of parkinsonian syndromes in a
specialist movement disorders service. Brain.

[24] Hoehn MM, Yahr MD (2001). Parkinsonism: onset, progression, and mortality
1967. Neurology.

[25] Bruck SMD, Nitrini R, Caramelli P, Bertolucci PHF, Okamoto IH (2003). Suggestions for utilization of the mini-mental state examination
in Brasil. Arq Neuropsiquiatr.

[26] Sanches MAS, Lourenço RA (2009). Informant Questionnaire on Cognitive Decline in the Elderly
(IQCODE): adaptação transcultural para uso no
Brasil. Cad Saúde Pública.

[27] Mahler LA, Ramig LO, Fox C (2015). Evidence-based treatment of voice and speech disorders in
Parkinson's disease. Curr Opin Otolaryngol Head Neck Surg.

[28] Wight S, Miller N (2015). Lee Silverman Voice Treatment for people with Parkinson's: audit
of outcomes in a routine clinic. Int J Lang Commun Disord.

[29] Maillet A, Krainik A, Debu B (2012). Levodopa effects on hand and speech movements in patients with
Parkinson's disease: a fMRI study. PLos One.

[30] Dromey C, Ramig LO (1998). The effect of lung on selected phonatory and articulatory
variables. J Speech Lang Hear Res.

[31] Baumgartner C, Sapir S, Ramig LO (2001). Perceptual voice quality changes following phonatory-respiratory
effort treatment (LSVT^®^) vs respiratory effort treatment
for individuals with Parkinson disease. J Voice.

[32] Sapir S, Spielman JL, Ramig LO, Story BH, Fox C (2007). Effects of intensive voice treatment (the Lee Silverman Voice
Treatment [LSVT^®^]) on vowel articulation in
dysarthric individuals with idiopathic Parkinson disease: acoustic and
perceptual findings. J Speech Lang Hear Res.

[33] Darling M, Huber JE (2011). Changes to articulatory kinematics in response to loudness cues
in individuals with Parkinson's disease. J Speech Lang Hear Res.

[34] Constantinescu G, Theodoros D, Russell T, Ward E, Wilson S, Wootton R (2011). Treating disordered speech and voice in Parkinson's disease
online: a randomized controlled non-inferiority trial. Int J Lang Commun Disord.

[35] Dias AE, Limongi JCP, Hsing WT, Barbosa ER Telemedicina para reabilitação da voz na doença de
Parkinson.

[36] Mahler LA, Ramig LO, Fox C (2015). Evidence-based treatment of voice and speech disorders in
Parkinson disease. Curr Opin Otolaryngol Head Neck Surg.

[37] Wight S, Miller N (2015). Lee Silverman Voice Treatment for people with Parkinson's: audit
of outcomes in a routine clinic. Int J Lang Commun Disord.

[38] Ramig L, Countryman S, O'Brien C, Hoehn M, Thompson L (1996). Intensive speech treatment for Parkinson's disease: short-and
long-term. Neurology.

[39] Ramig L, Sapir S, Fox C, Countryman S (2001). Changes in vocal intensity following intensive voice treatment
(LSVTÒ) in individuals with Parkinson disease. A comparison with
untreated patients and normal age-matched controls. Mov Disord.

[40] Molini-Avejonas DR, Rondon-Melo S, Amato CA, Samelli AG (2015). A systematic review of the use of telehealth in speech language
and hearing sciences. J Telemed Telecare.

[41] Dias A, Limongi J, Hsing WT, Barbosa ER (2013). Cognition and education to speech telerehabilitation in
Parkinson's disease. Abstract Book.

[42] Choi NG, Dinitto DM (2013). Internet use among older adults: association with health needs,
psychological capital, and social capital. J Med Internet Res.

[43] Achey MA, Beck CA, Beran DB (2014). Virtual house calls for Parkinson's disease (Connect.Parkinson):
study protocol for a randomized, controlled trial. Trials.

[44] Meyer A, Zimmermann R, Gschwandter U (2015). Apathy in Parkinson's disease is related to executive function,
gender and age but nor to depression. Front Aging Neurosci.

[45] Bertollucci PH, Brucki SM, Campacci SR, Juliano Y (1994). The mini-Mental State Examination in a general population: impact
of educational status. Arq Neuropsiquiatr.

[46] Robbins TW, Cools R (2014). Cognitive deficits in Parkinson's disease: a cognitive
neuroscience perspective. Mov Disord.

[47] Dubois B, Pillon B (1997). Cognitive deficits in Parkinson's disease. J Neurol.

[48] Beijer LJ, Rietveld TC, Hoskam V, Geurts AC, de Swart BJ (2010). Evaluating the feasibility and the potential efficacy of
e-learning-based speech therapy (EST) as a web application for speech
training in dysarthric patients with Parkinson's disease: a case
study. Telemed J E Health.

[49] Hailey D, Roine R, Ohinmaa A, Dennett L (2011). Evidence of benefit from telerehabilitation in routine care: a
systematic review. J Telemed Telecare.

[50] Theodoros D (2012). A new era in speech-language pathology practice: innovation and
diversification. Int J Speech Lang Pathol.

